# Clinical epidemiology of herpes zoster and postherpetic neuralgia in Barcelona (Spain). A single-center study

**DOI:** 10.3389/fpubh.2026.1743321

**Published:** 2026-02-17

**Authors:** Montserrat Salleras, Patricia Salvador, Lluís Salleras, Núria Soldevila, Andreu Prat, Patricio Garrido, Angela Domínguez

**Affiliations:** 1 Dermatology Service, Hospital del Sagrado Corazón, Barcelona, Spain; 2 Department of Medicine, Universitat de Barcelona, Barcelona, Spain; 3 CIBER Epidemiología y Salud Pública (CIBERESP), Instituto de Salud Carlos III, Madrid, Spain

**Keywords:** neuralgia, older adults, pain severity, risk factors, shingles

## Abstract

**Introduction:**

Varicella-zoster virus is an alphaherpesvirus that causes two diseases in humans: chickenpox, the primary infection, and herpes zoster (HZ). Although HZ incidence has been well-documented, relatively little is known regarding the clinical-epidemiological characteristics of HZ prodromal pain, acute pain, and subacute pain. The aim of the study was to analyse the clinical-epidemiological characteristics of HZ and postherpetic neuralgia (PHN) in cases diagnosed between 2007 and 2020.

**Methods:**

We conducted a clinical-epidemiological study of cases of HZ and PHN diagnosed between 2007 and 2020 in Hospital de Sagrado Corazón (Barcelona) in the first 14 days after rash onset, analyzing age, gender, dermatomes, and pain. Pain on rash diagnosis was classified as no pain, any pain, and clinically significant pain (scores 0, 1–10, and 3–10, respectively).

**Results:**

A total of 589 cases of HZ were included, mean age of patients was 59.6 years and the female/male ratio was 1.38 and cases increased with age (*p* < 0.01). Of the 589 cases, 77.4% had pain on day 0 of rash onset, 34.5% at 30 days, and 11.9% at 90 days. 70 cases progressed to PHN, which also increased with age. Factors associated with PHN were age groups 70–79 years (aOR 4.26; 95% CI 1.36–13.37) and ≥80 years (aOR 3.89; 95% CI 1.18–12.87) and intensity of pain score at rash onset ≥7 (aOR 19.74; 95% CI 4.63–84.10).

**Conclusions:**

Patient age and pain presence and intensity on day 0 and at 30 days of HZ diagnosis are the main risk factors for PHN.

## 1 Introduction

Varicella-zoster virus (VZV) is an alphaherpesvirus that causes two diseases in humans: chickenpox, the primary infection, and herpes zoster (shingles), caused by the endogenous reactivation of viruses that persist latently in the cranial and spinal sensory root ganglia after primary infection (1–3).

In Barcelona (Catalonia, Spain), as in developed countries, VZV infection especially affects very young children (4, 5). Before the introduction of the varicella vaccination, 82, 92, and 94% of children aged 5–9, 10–14, and 15–34 years became infected, and nearly all persons aged ≥50 years were infected (4, 5). This means that nearly 100% of the adult and elderly population of Barcelona harbor VZV in their sensorial ganglia, and this situation persists as long as cellular immunity against the virus is maintained. If immunity wanes, due to a medical or environmental condition that affects cellular immunity or due to age-related immunosenescence, the virus may affect the skin and cause herpes zoster (HZ) (2, 6). Latent infection occurs in most primary VZV infection cases, but only around 30% of infected persons will develop HZ at some point in their life, while 50% of persons who reach the age of 85 years without HZ will develop HZ (6, 7).

In Catalonia, the varicella vaccine was included in the recommended immunization schedule in 2005 with two doses at age 12 years. In 2016 it was changed to doses at 15 months and 3 years. Herpes zoster vaccine was included in 2022 in the recommended immunization schedule with 2 doses for people aged 65 or 80 years. Varicella vaccination is highly effective, with the transition to 2-dose schedules significantly reducing varicella incidence without increasing herpes zoster rates as initially hypothesized by some authors (8, 9). Both varicella and herpes zoster vaccines have demonstrated favorable safety profiles, including the combined MMRV vaccine (10) and the more recent recombinant adjuvanted herpes zoster vaccine, even in high-risk immunocompromised populations (11).

Clinically, HZ is characterized by a vesicular eruption of unilateral metameric distribution, in many—but not all—cases accompanied by pain, and limited in most cases to the dermatome innervated by a single sensory cranial or spinal ganglion, although the disease can also affect several dermatomes at the same time (2, 3). In many cases, rash onset is preceded by pain, paraesthesia, itching, and other forms of neurological discomfort in the involved dermatome, lasting several days and sometimes a week or more (2, 3, 12). This prodromal pain is only diagnosed when the rash develops, and consequently, other pain-causing diseases are frequently suspected, leading to numerous primary care and emergency department visits (2, 12).

HZ is a common disease, with similar annual incidence in most countries (four per 1,000 inhabitants in Europe). HZ incidence increases with age and mainly affects older people and women (6, 7, 12–15), and many patients have risk factors and underlying chronic diseases (3, 13, 15). Specific antiviral treatments are available, but even if administered immediately after rash onset, they rarely prevent complications, the most serious of which are postherpetic neuralgia (PHN) and ocular conditions (3, 14). In recent years, HZ incidence has been increasing (15–19), attributed to population aging and the emergence of new treatments for chronic diseases that lead to immunosuppression (16–19). All in all, HZ constitutes an important public health problem (15, 16), as the pain experienced represents a significant burden for the healthcare system and affects the patient's quality of life (12, 13).

In addition to its clinical impact, herpes zoster and postherpetic neuralgia are associated with a substantial economic burden. An economic burden study of herpes zoster in Spain reported that the disease generates substantial direct and indirect costs, significantly higher in patients with NPH (20), supporting the relevance of herpes zoster and PHN as a major public health concern.

Since the availability of attenuated vaccines (21) and inactivated vaccines (22) (2006 and 2016, respectively), there has been extensive research into HZ epidemiology and vaccination cost-effectiveness. Although HZ incidence has been well-documented (23–25), relatively little is known regarding the clinical-epidemiological characteristics of HZ prodromal pain, acute pain (0 days after rash onset), and subacute pain (30–90 days after rash onset).

The aim of the study was to analyse the clinical-epidemiological characteristics of HZ and PHN in cases diagnosed between 2007 and 2020.

## 2 Materials and methods

Included in the case series were all HZ and PHN diagnoses carried out during the years 2007 and 2020 within the first 14 days after rash onset in dermatology department and outpatient clinics of Hospital del Sagrado Corazón. The Hospital del Sagrado Corazón is a hospital located in Barcelona, Spain, with approximately 350 inpatient beds, which annually attend to approximately 24,000 hospital discharges, 52,000 emergencies and more than 350,000 outpatient visits.

HZ was defined as a vesicular rash with a unilateral metameric distribution, usually presenting with pain at the eruption site. The diagnosis was clinical in almost all cases, with the polymerase chain reaction (PCR) test reserved for doubtful cases (26). Prodromal pain was defined as any neurological pain or discomfort at the HZ eruption site before rash onset. PHN was defined as a unilateral metameric pain that persists for 90 days after rash onset.

Pain and discomfort related to the prodromal, acute, and subacute phases of HZ and PHN were measured according to Zoster Brief Pain Inventory criteria (21, 27), which, using an 11 point scale (0 = no pain to 10 = pain as bad as can be imagined), measures the severity of current, least, worst, and mean pain and discomfort in the previous 24 h. This instrument, used in the Shingles Prevention Study,16 has been widely demonstrated to have good reliability and validity.

An initial diagnostic visit was conducted, followed by biweekly follow-up visits until healing was achieved. If clinically significant pain persisted, PHN follow-up was extended to 18 months.

In our research, pain was “the worst pain” experienced by the patient in the 24 h prior to each dermatology visit and to enable comparison of our pain indicators with those of other studies, we used two criteria: “any pain” and “clinically significant pain” (scored 1–10 and 3–10, respectively). HZ and PHN cases were also recorded as classically diagnosed, i.e., without taking pain into account in the case definition.

On day 0 of rash diagnosis, HZ cases were classified into three categories: no pain (score 0), any pain (score 1–10), and clinically significant pain (score 3–10). PHN cases were classified in two groups: any pain (score 1–10) and clinically significant pain (score 3–10).

Collected variables included age, sex, eruption site, presence of pain, pain intensity at diagnosis and during follow-up (day 0, day 30, and day 90), duration of pain, immune suppressive disease or therapy, comorbidities and antiviral treatment. Clinical and epidemiological data were collected prospectively from patients' hospital visits.

Bivariate comparisons were performed using Pearson's chi-square test for categorical variables, and trend was assessed using the chi-square trend test. Crude odds ratio (OR), adjusted OR (aOR), and 95% CI values were estimated using logistic regression. Adjusted OR values were calculated using the logistic regression model with backward variable selection, with a cut-off point of *p* < 0.2. Differences were considered statistically significant for *p* < 0.05.

### 2.1 Ethical considerations

The data were collected retrospectively in December 2024. All data collected from patients were treated as confidential, in strict observance of the legislation on observational studies. The study was approved by the Hospital del Sagrado Corazón ethics committee (2024/92-DER-HUSC).

## 3 Results

589 cases of HZ were included, 342 (58.1%) were women and median was 63 (6–99) years. Table 1 describes HZ cases by age and gender according to the two pain categories (any pain and clinically significant pain). Women compared to men predominated overall, although men predominated in the < 20 age groups. The female/male ratio overall was 1.38, and was slightly greater in both the any pain (1.53) and clinically significant pain (1.57) categories.

**Table 1:** Herpes zoster cases by pain category classified by age and gender.

**Age, years**	**Overall**	**Men**	**Women**	**Female/male ratio**
**Overall**				
Median (range)	63 (6–99)	64 (6–89)	63 (7–99)	
0–9	13 (2.2%)	8 (3.2%)	5 (1.5%)	0.62
10–19	37 (6.3%)	24 (9.7%)	13 (3.8%)	0.54
20–29	16 (2.7%)	6 (2.4%)	10 (2.9%)	1.67
30–39	30 (5.1%)	10 (4.0%)	20 (5.8%)	2.00
40–49	55 (9.3%)	17 (6.9%)	38 (11.1%)	2.23
50–59	96 (16.3%)	36 (14.6%)	60 (17.5%)	1.67
60–69	118 (20.0%)	46 (18.6%)	72 (21.1%)	1.56
70–79	120 (20.4%)	56 (22.7%)	64 (18.7%)	1.14
≥80	104 (17.7%)	44 (17.8%)	60 (17.5%)	1.36
All ages	589	247	342	1.38
**Any pain**				
Mean ± SD	66 (7–99)	68 (7–89)	65 (11–99)	
0–9	4 (0.9%)	4 (2.2%)	0 (0.0%)	0.00
10–19	9 (2.0%)	6 (3.3%)	3 (1.1%)	0.50
20–29	6 (1.3%)	3 (1.7%)	3 (1.1%)	1.00
30–39	21 (4.6%)	7 (3.9%)	14 (5.1%)	2.00
40–49	38 (8.3%)	10 (5.6%)	28 (10.1%)	2.80
50–59	81 (17.8%)	29 (16.1%)	52 (18.8%)	1.79
60–69	101 (22.1%)	38 (21.1%)	63 (22.8%)	1.66
70–79	108 (23.7%)	49 (27.2%)	59 (21.4%)	1.20
≥80	88 (19.3%)	34 (18.9%)	54 (19.6%)	1.59
All ages	456	180	276	1.53
**Clinically significant pain**				
Mean ± SD	67 (7–99)	68 (7–89)	66 (11–99)	
0–9	3 (0.7%)	3 (1.9%)	0 (0%)	0.00
10–19	8 (2.0%)	5 (3.2%)	3 (1.2%)	0.60
20–29	4 (1.0%)	2 (1.3%)	2 (0.8%)	1.00
30–39	19 (4.7%)	6 (3.8%)	13 (5.3%)	2.17
40–49	31 (7.7%)	8 (5.1%)	23 (9.3%)	2.87
50–59	73 (18.1%)	27 (17.2%)	46 (18.7%)	1.70
60–69	86 (21.3%)	31 (19.7%)	55 (22.4%)	1.77
70–79	99 (24.6%)	45 (28.7%)	54 (22.0%)	1.20
≥80	80 (19.9%)	30 (19.1%)	50 (20.3%)	1.67
All ages	403	157	246	1.57

HZ diagnoses clearly increased with age (*p* < 0.01), irrespective of the pain category. The lowest number of cases occurred in children aged 0–9 years and adults aged 20–29 years, and the highest number of cases occurred in the 70–79 age group (followed by the 60–69 age group), after which cases dropped in the ≥80 age group.

Table 2 shows eruption site details for the < 50 years and ≥50 age groups. Overall, the thoracic dermatome (35.0%) was most affected, followed at a distance by the lumbar (19.8%), cranial (18%), sacral (14.6%), and cervical (11.9%) dermatomes. For both any pain and clinically significant pain, proportions were higher in the ≥50 age group, with the exception of the thoracic and cranial dermatomes, for which proportions were higher in the < 50 age group. Disseminated cases accounted for 0.7% of patients, and there were no cases of HZ sine herpete.

**Table 2:** Herpes zoster cases by eruption site classified by age group.

**Eruption site**	**Overall (*N* = 589)**	** < 50 years (*N* = 151)**	**≥50 years (*N* = 438)**	***p*-value**
**Overall**				
Thoracic	206 (35.0%)	67 (44.3%)	139 (31.7%)	Ref.
Lumbar	117 (19.8%)	27 (17.9%)	90 (20.6%)	0.07
Cranial	106 (18.0%)	19 (12.6%)	87 (19.9%)	0.01
Cervical	70 (11.9%)	17 (11.3%)	53 (12.1%)	0.20
Sacral	86 (14.6%)	21 (13.9%)	65 (14.8%)	0.17
Disseminated	4 (0.7%)	0 (0%)	4 (0.9%)	0.99
**Any pain (score 1–10)**				
Thoracic	153 (33.6%)	29 (37.2%)	124 (32.8%)	Ref.
Lumbar	91 (20.0%)	15 (19.2%)	76 (20.1%)	0.63
Cranial	92 (20.2%)	17 (21.8%)	75 (19.8%)	0.93
Cervical	52 (11.4%)	8 (10.3%)	44 (11.6%)	0.56
Sacral	65 (14.3%)	9 (11.5%)	56 (14.8%)	0.36
Disseminated	3 (0.7%)	0 (0%)	3 (0.8%)	0.99
**Clinically significant pain (score 3–10)**				
Thoracic	139 (34.5%)	26 (40.0%)	113 (33.4%)	Ref.
Lumbar	81 (20.1%)	12 (18.5%)	69 (20.4%)	0.46
Cranial	74 (18.4%)	13 (20.0%)	61 (18.0%)	0.84
Cervical	49 (12.2%)	7 (10.8%)	42 (12.4%)	0.49
Sacral	58 (14.4%)	7 (10.8%)	51 (15.1%)	0.26
Disseminated	2 (0.5%)	0 (0%)	2 (0.6%)	0.99

Table 3, summarizes pain at different HZ stages (day 0, at 30 days, and at 90 days) according to age group and pain category. On day 0, 133 patients (22.6%) had no pain, and the proportion of patients aged < 50 years with no pain (48.3%) was much higher than for patients aged ≥50 years (13.7%). For any pain, the percentage of cases on day 0 was 77.4%, decreasing to 34.5% at 30 days, and 11.9% at 90 days; percentages for clinically significant pain were slightly lower at 68.4% on day 0, 31.6% at 30 days, and 11.4% at 90 days. For both the pain categories and for all measurements, pain percentages for the < 50 age group were much lower than for the ≥50 age group (*p* < 0.01).

**Table 3:** Herpes zoster cases by pain category at different disease stages classified by age group.

**Disease stage**	**Overall (*N* = 589)**	** < 50 years (*N* = 151)**	**≥50 years (*N* = 438)**	***p*-value**
No pain (score 0)	133 (22.6%)	73 (48.3%)	60 (13.7%)	
Pain day 0	456 (77.4%)	78 (51.7%)	378 (86.3%)	
**Any pain (score 1–10)**				
Day 0 of rash onset	456 (77.4%)	78 (51.7%)	378 (86.3%)	< 0.01
Day 30 of rash onset	203 (34.5%)	18 (11.9%)	185 (42.3%)	< 0.01
Day 90 of rash onset	70 (11.9%)	4 (2.6%)	66 (15.1%)	< 0.01
**Clinically significant pain (score 3–10)**				
Day 0 of rash onset	403 (68.4%)	65 (43.0%)	338 (77.2%)	< 0.01
Day 30 of rash onset	186 (31.6%)	15 (9.9%)	171 (39.0%)	< 0.01
Day 90 of rash onset	67 (11.4%)	4 (2.6%)	63 (14.4%)	< 0.01

Table 4 summarize patterns according to age, revealing age distribution patterns that were similar for both pain categories, although proportions were slightly lower for the clinically significant pain category compared to the any pain category.

**Table 4:** Herpes zoster pain characteristics by age at different disease stages.

**Characteristics**	**Overall *N* = 589**	**Day 0 *N* = 456**	**Day 30 *N* = 203**	**Day 90 *N* = 70**	*p*-value^*^	*p*-value^**^
**Any pain (score 1–10)**						
With pain	456 (77.4%)	203 (34.5%)	70 (11.9%)			
<50 years	151	78 (51.6%)	18 (11.9%)	4 (2.6%)	0.01	0.44
50–59 years	96	81 (84.4%)	29 (30.2%)	8 (8.3%)	0.23	0.60
60–69 years	118	101 (85.6%)	42 (35.6%)	12 (10.2%)	0.41	0.58
70–79 years	120	108 (90.0%)	68 (56.7%)	26 (21.7%)	0.04	0.65
≥80 years	104	88 (84.6%)	46 (44.2%)	20 (19.2%)	0.13	0.39
≥50 years	438	378 (86.3%)	185 (42.2%)	66 (15.1%)	0.33	0.81
Median pain severity	6.5 (1–10)	6 (1–10)	5.3 (1–10)			
**Pain intensity (score)**						
Mild (1–2)		50 (11.0%)	17 (8.5%)	7 (10.0%)	0.84	0.71
Moderate (3–6)		178 (39.3%)	103 (51.8%)	42 (60.0%)	0.02	0.42
Severe (≥7)		225 (49.7%)	79 (39.7%)	21 (30.0%)	0.02	0.25
**Clinically significant pain (score 3–10)**						
With pain		403 (68.4%)	183 (31.1%)	63 (10.7%)		
< 50 years	151	65 (43.0%)	13 (8.6%)	3 (2.0%)	0.03	0.57
50–59 years	96	73 (76.0%)	27 (28.1%)	7 (7.3%)	0.21	0.52
60–69 years	118	86 (72.9%)	38 (32.2%)	10 (8.5%)	0.37	0.46
70–79 years	120	99 (82.5%)	64 (53.3%)	24 (20.0%)	0.05	0.71
≥80 years	104	80 (76.9%)	41 (39.4%)	19 (18.3%)	0.10	0.29
≥50 years	438	338 (77.2%)	170 (38.8%)	60 (13.7%)	0.36	0.86
Median pain severity	7 (3–10)	6 (3–10)	6 (3–10)			
**Pain intensity (score)**						
Moderate (3–6)		178 (44.2%)	104 (56.8%)	42 (66.7%)	0.02	0.38
Severe (≥7)		225 (55.8%)	79 (43.2%)	21 (33.3%)	0.02	0.29

^*^Postherpetic neuralgia vs. pain day 0; ^**^Postherpetic neuralgia vs. pain day 30.

Pain at all stages increased progressively through the different age groups, peaking in the 70–79 age group and falling off thereafter. On day 0, for instance, any pain was 51.6% for the < 50 age group, peaked at 90% for the 70–79 age group, and fell to 84.6% for the ≥80 age group. Median pain severity was 6.5 (1–10), and 49.7, 39.3, and 11% of patients reported severe, moderate, and mild pain, respectively. The severe pain rate was significantly lower at 30 days (39.7%) than on day 0 (49.7%). Of the included cases, just 70 (11.9%) had any pain 90 days after rash onset, and their median pain severity was lower than on day 0 (5.3 vs. 6.5). The pain percentage at 90 days for patients aged 70–79 years (the age group with the highest incidence) was much lower than on day 0 (21.7% vs. 90%; *p* = 0.04). For clinically significant pain, the severe pain rate was significantly lower at 90 days (33.3%) than on day 0 (55.8%); *p* = 0.02.

Table 5 summarizes factors associated with PHN. Of the 589 cases, 70 (11.9%) progressed to PHN with a median age of 74 (44–91) years. Factors associated with PHN were age groups 70–79 years (aOR 4.26; 95% CI 1.36–13.37) and ≥80 years (aOR 3.89; 95% CI 1.18–12.87) and intensity of pain score at rash onset ≥7 (aOR 19.74; 95% CI 4.63–84.10).

**Table 5:** Factors associated with PHN in patients with herpes zoster.

**Characteristics**	**PHN**	**HZ without PHN (*N* = 519)**	**OR (IC 95%)**	***p*-value**	**aOR (IC 95%)**	***p*-value**
**Age**						
< 50 years	4 (2.6%)	147 (97.4%)	Ref.		Ref.	
50–59 years	8 (12.1%)	88 (23.6%)	3.34 (0.98–11.42)	0.05	1.23 (0.32–4.71)	0.76
60–69 years	12 (18.2%)	105 (28.5%)	4.16 (1.31–13.26)	0.02	2.05 (0.60–6.97)	0.25
70–79 years	26 (39.4%)	94 (25.3%)	10.16 (3.44–30.05)	< 0.01	4.26 (1.36–13.37)	0.01
≥80 years	20 (30.3%)	84 (22.6%)	8.75 (2.89–26.46)	< 0.01	3.89 (1.18–12.84)	0.03
**Sex**						
Male	30 (42.9%)	217 (41.8%)	Ref.			
Female	40 (57.1%)	302 (58.2%)	0.96 (0.58–1.59)	0.87		
**Presence of pain at rash onset**						
Yes	70 (100%)	386 (74.4%)	–	< 0.01		
No	0 (0%)	133 (25.6%)	Ref.			
**Pain score at rash onset**						
No or 1–2	3 (4.3%)	180 (34.9%)	Ref.		Ref.	
3–6	9 (12.9%)	169 (32.8%)	3.20 (0.85–12.00)	0.08	3.88 (0.81–18.49)	0.09
≥7	58 (82.9%)	167 (32.4%)	20.84 (6.41–67.78)	< 0.01	19.74 (4.63–84.10)	< 0.01
**Eruption site**						
Thoracic	20 (28.6%)	186 (35.8%)	0.72 (0.41–1.24)	0.23		
Other	50 (71.4%)	333 (64.2%)	Ref.			
**Pre-existing medical conditions**						
Yes	27 (38.6%)	166 (32.0%)	1.34 (0.80–2.24)	0.27		
No	43 (61.4%)	353 (68.0%)	Ref.			
**Immune suppressive disease or therapy**						
Yes	4 (5.7%)	35 (6.7%)	0.84 (0.29–2.43)	0.74		
No	66 (94.3%)	484 (93.3%)	Ref.			
**Comorbidities**						
Autoimmune disease	2 (2.1%)	11 (2.1%)	1.36 (0.29–6.26)	0.69		
Chronic respiratory disease	1 (1.4%)	8 (1.5%)	0.93 (0.11–7.51)	0.94		
Cardiovascular disease	8 (11.4%)	37 (7.1%)	1.68 (0.75–3.77)	0.21		
Chronic renal disease	4 (5.7%)	12 (2.3%)	2.56 (0.80–8.17)	0.11		
Diabetes mellitus	2 (2.9%)	37 (7.1%)	0.38 (0.09–1.63)	0.19	0.29 (0.07–1.31)	0.11
Depression/anxiety/stress	5 (7.1%)	39 (7.5%)	0.95 (0.36–2.49)	0.91		
Hypothyroidism	7 (10.0%)	47 (9.1%)	1.12 (0.48–2.58)	0.80		
**Antiviral treatment**						
Yes	54 (90.0%)	335 (70.7%)	3.73 (1.57–8.88)	0.01		
No	6 (10.0%)	139 (29.3%)	Ref.			

Pain duration was 3–6 months for 56.1%, 7–12 months for 25.8%, 13–18 months for 6.1%, and >18 months for 12.1% of patients aged ≥50 with PHN.

## 4 Discussion

Our results confirm that women are at higher risk of developing HZ than men. In our study, HZ was more frequent in women in all age groups except for the youngest age groups (children and young people aged < 20 years), in which males predominated. For all the cases included in our series, the female/male ratio overall was 1.38, considerably higher than that of the entire population of Barcelona (1.05) in 2012 (halfway through the study period) (28); furthermore, this ratio increased even further for the any pain (1.53) and clinically significant pain (1.57) categories.

The vast majority of studies published in the last 20 years have reported statistically significant higher HZ rates in women compared to men. A meta-analysis by Kawai et al. (29), which included 27 studies with different designs carried out on five continents in the last 18 years, reported that women were consistently at greater risk of developing HZ than men (pooled effect estimate: relative risk (RR) 1.31; 95% confidence interval (CI) 1.27–1.34). The pooled effect estimate was somewhat lower in the meta-analysis by Marra et al. (30), which included 56 studies (RR 1.19; 95% CI 1.14–1.24). In another systematic review it was found that the female/male ratio of HZ cases was 1.1 (31). A study carried out in Spain found that HZ was more frequent in women than in men (32), and in a study carried out in China, women had a slightly higher incidence rate than men (7.9 vs. 7.6%) (33).

Some authors suggest—possibly implausibly—that the gender differences may be due to women's greater healthcare-seeking behavior. However, gender differences similar to those of the above-mentioned meta-analyses were reported for the placebo group of the Oxman et al. study (21), based on PCR diagnosis and exhaustive follow-up for 3 years. Our results pointing to a higher rate of HZ in males aged < 20 years compared to adults aged ≥20 years (predominantly women) would suggest an endogenous difference between males and females, probably of a hormonal or immunological nature (30, 34).

For many years, before the chickenpox vaccine was included in routine vaccination schedules, it was hypothesized that cellular immunity in the adult population would be periodically reinforced by viral exposure through contact with children (35). If this were the case, since it is women who usually care for sick children, female cellular immunity would be strengthened relative to that of males; consequently, incidence of HZ in women would be lower and would require less immunity boosting. Our results, however, would point to a rejection of this hypothesis, as does a Japanese study of pediatricians who theoretically would be more exposed to the virus (36), and as does a French study of cloistered nuns with no relationship with children over their entire lives (37).

Our study also confirms the increase in HZ incidence with age, corroborating most other similar studies. A systematic review found than age was identified as a major risk factor toward HZ incidence which increase significantly in people >50 years of age (31). Yang et al. (33) found that rates of HZ increased with age and were highest in patients aged ≥70 years and Masa-Calles et al. (32) also found that the incidence of HZ increased with age. The explanation for the age association, better grounded than that of the gender association, is cell immunosenescence linked to aging and to diseases that depress immune systems (38–41). As reported in most other similar studies, in our study, incidence peaked in the 70–79 age group and declined from age 80 onwards. There is no clear explanation for this decline in the elderly, as cellular immunity continues to decrease after the age of 80 years. Some authors hypothesize that illness in this age group is less likely to be recorded in official statistics, given the higher prevalence of disabling chronic diseases that hinder mobility and so lead to more home-based treatment by family doctors rather than in outpatient clinics or primary care centers (39, 42, 43).

In our study, and corroborating most other studies, the thoracic dermatome was most affected, followed at a distance by the lumbar dermatome, and then—contrary to most studies (41–45)—by the cranial dermatome, probably because patients with ocular involvement, alarmed by dramatic signs and symptoms, go directly to the ophthalmologist rather than to the dermatologist. Some authors have posited a possible role for specific dermatomes, most especially the trigeminal dermatome, in PHN development, although a meta-analysis by Forbes et al. (45) was unable to conclusively demonstrate this role. In our study, distribution according to the affected dermatome of HZ cases with progression to PHN was similar to that for non-progression to PHN, which would suggest that rash site is not a risk factor for this complication.

To our knowledge, ours is the first study of the epidemiology of HZ and PHN carried out in Spain in the form of a single-center and single-dermatologist study. HZ cases have been classified in three pain categories: no pain (score 0), any pain (score 1–10), and clinically significant pain (score 3–10). Pain incidence on diagnosis was relatively high, but decreased significantly after 30 days of evolution, from 77.4 to 34.5% of patients. Noteworthy was the fact that the proportion of cases with significant clinically pain (3–10) was 68.4% at 0 days and 31.1% at 30 days compared to 77.4 and 34.5%, respectively, for any pain (1–10).

The incidence of pain on diagnosis clearly increased with age in our study, corroborating the majority of published studies (13, 15, 29, 30, 46). While the percentage of patients with pain on day 0 was relatively high (77.4%), it decreased significantly by 30 days (34.5%). However, mean pain intensity did not decrease in the same proportion over the same period, contrasting with other studies reporting that mean pain intensity decreased significantly between 0 and 30 days after rash onset (47–51).

PHN incidence, like that of HZ, clearly increased with age. For patients with HZ, the mean age of progression to PHN was significantly higher (72 years) than for patients with non-progression (58 years), reflecting a significant difference of 14 percentage points; this would suggest that age at diagnosis is an important risk factor for PHN in patients with HZ. Yang et al. (33) also found that rates of PHN increased with age. In a study carried out in Saudi Arabia it was found that a significant predictor of NPH was age ≥50 years (52).

Finally, noteworthy is the fact that PHN duration was 3–12 months in 81.9%, 13–18 months in 6.1%, and >18 months in 12.1% of patients aged ≥50 years (8 of the PHN cases continued to experience pain 18 months post-onset). Other authors have found similar values, most patients had PHN-related pain for < 1 year (80.5%), with only a small proportion having PHN-related pain for ≥5 years (4.3%) (33).

The main strength of our study is that all patients were diagnosed and registered by the same experienced dermatologist (Dr Salleras, director of our hospital dermatology department), thereby avoiding interpersonal variability, which avoided the biases that often occur in diagnoses and data transcription from primary healthcare or hospital records to computerized databases. In an epidemiological study in Olmsted County (USA), Yawn et al. (53) compared medical record review and administrative database estimates of HZ rates and complications, finding a difference in HZ occurrence of 14.5%; while differences of 15%−18% may be acceptable for incidence or trend studies, they are clearly excessive when greater precision is required, e.g., for economic studies. Other recently published studies have also found significant differences between administrative databases and medical records for case and vaccination data (54, 55).

A key strength differentiating our study from most similar studies is that patients of all ages (children, adolescents, young adults, and older adults) were included. Many studies published since vaccines became available include persons who are the usual target of vaccination programmes, i.e., aged ≥50 years in whom the HZ incidence is higher. Our inclusion of a broader range of ages shows that HZ and PHN also affect people aged < 50 years, and also confirms less severe clinical characteristics compared to persons aged ≥50 years.

Another strength is that pain intensity data was recorded by the same specialist, reflecting a single criterion in the interpretation of pain. Moreover, incidence was calculated using same definitions from recently published studies, i.e., three categories for HZ and two categories for PHN, thereby enabling comparison with other studies that use the same definitions.

Since our study was based on data for patients with HZ and PHN visiting mostly a dermatology outpatients clinic, the included cases may be more severe than those treated in primary care. Likewise, the fact that the subjects included in the study were healthcare-seeking patients likely explains the greater severity in our series compared to experimental studies implemented to evaluate the two marketed vaccines. In those latter studies, cases were detected among persons randomly included in the vaccinated and control groups, and consequently, those with mild HZ who did not seek medical attention would have been excluded.

Regarding study limitations, other than for PHN in HZ cases, HZ incidence and complication rates could not be calculated, since 70% of persons treated by the dermatology outpatient clinic were referred from attached primary care centers, while the remaining 30% were referrals, according to the criteria of insurance companies, of residents of other areas of Barcelona and even of Catalonia. A further limitation (inherent to all epidemiology studies) is our non-inclusion of catchment-area patients who did not seek medical care for a mild form of HZ or who sought private care.

Finally, rash lesions have only been counted since 2021, so the data available on this variable are currently insufficient to draw statistically significant conclusions. Likewise, prodromal pain data have only been collected since 2014, resulting in a relatively small sample size and reducing the probability of detecting statistically significant differences in any possible associations.

During the study period, the herpes zoster vaccine was not available in the immunization schedule of Catalonia. Real-world evidence demonstrates that recombinant zoster vaccine safety and provides protection in high-risk populations (56), and a reduction in herpes zoster incidence among immunocompromised patients (57, 58). Future studies should incorporate vaccination status to better evaluate the impact of herpes zoster vaccination campaigns, particularly in frail and immunocompromised populations who benefit most from preventive strategies.

Despite the availability of antiviral therapy, the management and prevention of postherpetic neuralgia remain limited, with current treatments showing modest effectiveness. Recently, a case report described rapid symptom resolution and a shortened duration of PHN in a patient with severe herpes zoster ophthalmicus treated with a combination of an immunostimulatory vaccine virus and acyclovir (59). Although based on a single case, these findings highlight the potential of novel combination therapies and the need for further research into more effective treatment strategies for PHN.

Our study would confirm, as other authors have found (60), that pain presence and intensity at 0 and 30 days after HZ diagnosis and the patient's age are the main risk factors for PHN development. In recent years it has been observed that 14-day famciclovir administration and combined high-voltage radiofrequency and oxygen-ozone treatment may have some therapeutic and perhaps also preventive benefits in managing PHN. Our findings, if confirmed by other studies, could be used in clinical diagnosis to select patients who are more likely to develop PHN and who would benefit most from treatment.

## Data Availability

The original contributions presented in the study are included in the article, further inquiries can be directed to the corresponding author.

## References

[1] Hope-Simpson RE. The nature of herpes zoster: a long term study and a new hypothesis. Proc R Soc Med. (1965) 58:9–20. doi: 10.1177/003591576505800106 14267505 PMC1898279

[2] Schmader KE Dworkin RH. The epidemiology and natural history of herpes zoster and postherpetic neuralgia. In:Watson CPM Gershon AA Oxman MN, editors. Herpes Zoster: Postherpetic Neuralgia and Other Complications: Focus on Treatment and Prevention. Cham: Springer (2017) p. 25–44. doi: 10.1007/978-3-319-44348-5_4

[3] Johnson RW Alvarez-Pasquin MJ Bijl M Franco E Gaillat J Clara JG . Herpes zoster epidemiology, management, and disease and economic burden in Europe: a multidisciplinary perspective. Ther Adv Vaccines. (2015) 3:109–20. doi: 10.1177/2051013615599151 26478818 PMC4591524

[4] Salleras L Domínguez A Vidal J Plans P Salleras M Taberner JL. Seroepidemiology of varicella-zoster virus infection in Catalonia (Spain). Rationale for universal vaccination programmes. Vaccine. (2000) 19:183–8. doi: 10.1016/S0264-410X(00)00178-X 10930671

[5] Salleras L Domínguez A Plans P Costa J Cardeñosa N Torner N . Seroprevalence of varicella zoster virus infection in child and adult population of Catalonia (Spain). Med Microbiol Immunol. (2008) 197:329–33. doi: 10.1007/s00430-007-0064-z 18004592

[6] Bowsher D. The lifetime occurrence of herpes zoster and prevalence of post-herpetic neuralgia: a retrospective survey in an elderly population. Eur J Pain. (1999) 3:335–42. doi: 10.1016/S1090-3801(99)90015-0 10700361

[7] Lee C Giannelos N Curran D Dong H Tang H Jiang N . Lifetime risk of herpes zoster in the population of Beijing, China. Public Health Pract. (2023) 5:100356. doi: 10.1016/j.puhip.2023.100356 36968763 PMC10031117

[8] Wolfson LJ Daniels VJ Altland A Black W Huang W Ou W. The impact of varicella vaccination on the incidence of varicella and herpes zoster in the United States: updated evidence from observational databases, 1991–2016. Clin Infect Dis. (2020) 70:995–1002. doi: 10.1093/cid/ciz305 31147680

[9] Gil-Prieto R Walter S Gonzalez-Escalada A Garcia-Garcia L Marín-García P Gil-de-Miguel A. Different vaccination strategies in Spain and its impact on severe varicella and zoster. Vaccine. (2014) 32:277–83. doi: 10.1016/j.vaccine.2013.11.008 24275483

[10] Stefanizzi P De Nitto S Patano F Bianchi FP Ferorelli D Stella P . Post-marketing surveillance of adverse events following measles, mumps, rubella and varicella (MMRV) vaccine: retrospective study in Apulia region (ITALY), 2009-2017. Hum Vaccin Immunother. (2020) 16:1875–83. doi: 10.1080/21645515.2019.1704124 32040350 PMC7482746

[11] Stefanizzi P Moscara L Palmieri C Martinelli A Di Lorenzo A Venerito V . Safety profile of recombinant adjuvanted anti-herpes zoster vaccine (RZV) in high-risk groups: data from active surveillance program. Puglia (Italy), 2021-23. Vaccine. (2024) 42:2966–74. doi: 10.1016/j.vaccine.2024.03.024 38582693

[12] Kennedy PGE Gerhon AA. Clinical features of varicella-zoster virus infection. Viruses. (2018) 10:609. doi: 10.3390/v10110609 30400213 PMC6266119

[13] Pinchinat S Cebrián-Cuenca AM Bricout H Johnson RW. Similar herpes zoster incidence across Europe: results from a systematic literature review. BMC Infect Dis. (2013) 13:170. doi: 10.1186/1471-2334-13-170 23574765 PMC3637114

[14] Scott FT Johnson RW Leedham-Green M Davies E Edmunds WJ Breuer J. The burden of herpes zoster: a prospective population based study. Vaccine. (2006) 24:1308–14. doi: 10.1016/j.vaccine.2005.09.026 16352376

[15] Harpaz R Leung JW. The epidemiology of herpes zoster in United States during the era of varicella and herpes zoster vaccines: changing patterns among older adults. Clin Infect Dis. (2019) 69:341–4. doi: 10.1093/cid/ciy953 30496358

[16] Curran D Doherty TM Lecrenier N Breuer T. Healthy ageing: herpes zoster infection and de role of zoster vaccination. NPJ Vaccines. (2023) 8:184. doi: 10.1038/s41541-023-00757-0 38017011 PMC10684688

[17] Thompson RR Kong CL Porco TC Kim E Ebert CD Acharya NR. Herpez zoster and postherpetic neuralgia: changing incidence rates from 1994 to 2018 in the United States. Clin Infect Dis. (2021) 73:e3210–7. doi: 10.1093/cid/ciaa1185 32829399 PMC8563174

[18] Sodergen E Mardberg K Nishimw EM Bhavsar A Marijam A Berstrom Tand SP. Incidence and burden of herpes zoster in Sweden: a regional population -based register study. Infect Dis Ther. (2024) 13:121–40. doi: 10.1007/s40121-023-00902-1 38193987 PMC10828402

[19] Marziano V Poletti P Guzzetta G Ajelli M Manfredi P Merler S. The impact of demographic changes on the epidemiology of herpes zoster: Spain as a case study. Proc Biol Sci. (2015) 282:20142509. doi: 10.1098/rspb.2014.2509 25761709 PMC4375857

[20] Díez-Domingo J Curran D Cambronero MR Garcia-Martinez JA Matthews S. Economic burden and impact on quality of life of herpes zoster in Spanish adults aged 50 years or older: a prospective cohort study. Adv Ther. (2021) 38:3325–41. doi: 10.1007/s12325-021-01717-7 34013498 PMC8190024

[21] Oxman MN Levin MJ Johnson GR Schmader KE Straus SE Gelb LD . A vaccine to prevent herpes zoster and postherpetic neuralgia in older adults. N Engl J Med. (2005) 352:2271–84. doi: 10.1056/NEJMoa051016 15930418

[22] Lal H Cunningham AL Godeaux O Chlibek R Diez-Domingo J Hwang SJ . Efficacy of an adjuvanted herpes zoster subunit vaccine in older adults. N Engl J Med. (2015) 372:2087–96. doi: 10.1056/NEJMoa1501184 25916341

[23] Muñoz-Quiles C López-Lacort M Orrico-Sánchez A Díez-Domingo J. Impact of postherpetic neuralgia. A six years population-based analysis on people aged 50 years or older. J Infect. (2018) 77:131–6. doi: 10.1016/j.jinf.2018.04.004 29742472

[24] Cebrián-Cuenca AM Díez-Domingo J San-Martín-Rodríguez M Puig-Barberá J Navarro-Pérez J. Epidemiology and cost of herpes zoster and postherpetic neuralgia among patients treated in primary health care centers in the Valencian community of Spain. BMC Infect Dis. (2011) 11:302. doi: 10.1186/1471-2334-11-302 22044665 PMC3235981

[25] Salleras L Salleras M Salvador P Soldevila N Prat A Garrido P . Herpes zoster and postherpetic neuralgia in Catalonia (Spain). Hum Vaccin Immunother. (2015) 11:178–84. doi: 10.4161/hv.34421 25483532 PMC4514275

[26] Schremser V Antoniewicz L Tschachler E Geusau A. Polymerase chain reaction for the diagnosis of herpesvirus infections in dermatology: analysis of clinical data. Wien Klin Wochenschr. (2020) 132:35–41. doi: 10.1007/s00508-019-01585-w 31820101 PMC6978434

[27] Coplan PM Schmader K Nikas A Chan ISF Choo P Levin MJ . Development of a measure of the burden of pain due to herpes zoster and postherpetic neuralgia for prevention trials: adaptation of the brief pain inventory. J Pain. (2004) 5:344–56. doi: 10.1016/j.jpain.2004.06.001 15336639

[28] IDESCAT. Población a 1 de enero por edad y sexo. (2025). Spanish. Available online at: https://www.idescat.cat/pub/?geo=mun%3A080193&id=pmh&n=1180&lang=es#Plegable=geo (Accessed January 31, 2025).

[29] Kawai K Yawn BP. Risk factors for herpes zoster: a systematic review and meta-analysis. Mayo Clin Proc. (2017) 92:1806–21. doi: 10.1016/j.mayocp.2017.10.009 29202939

[30] Marra F Parhar K Huang B Vadlamudi N. Risk factors for herpes zoster infection: a meta-analysis. Open Forum Infect Dis. (2020) 7:ofaa005. doi: 10.1093/ofid/ofaa005 32010734 PMC6984676

[31] Bardach AE Palermo C Alconada T Sandoval M Balan DJ Nieto Guevara J . Herpes zoster epidemiology in Latin America: a systematic review and meta-analysis. PLoS ONE. (2021) 16:e0255877. doi: 10.1371/journal.pone.0255877 34383851 PMC8360515

[32] Masa-Calles J López-Perea N Vila Cordero B Carmona R. Surveillance and epidemiology of herpes zoster in Spain. Rev Esp Salud Pública. (2021) 95:e202106088. 34168107

[33] Yang F Yu S Fan B Liu Y Chen YX Kudel I . The epidemiology of herpes zoster and postherpetic neuralgia in China: results from a cross-sectional study. Pain Ther. (2019) 8:249–59. doi: 10.1007/s40122-019-0127-z 31218562 PMC6857181

[34] Fleming DM Cross KW Cobb WA Chapman RS. Gender differences in the incidence of shingles. Epidemiol Infect. (2004) 132:1–5. doi: 10.1017/S0950268803001523 14979582 PMC2870070

[35] Brisson M Edmunds WJ Law B Gay NJ Walld R Brownel M . Epidemiology of varicella zoster virus infection in Canada and the United Kingdom. Epidemiol Infect. (2001) 127:305–14. doi: 10.1017/S0950268801005921 11693508 PMC2869750

[36] Wu CY Hu HY Huang N Pu CY Shen HC Chou YY. Do the health-care workers gain protection against herpes zoster infection? A 6-year population-based study in Taiwan. J Dermatol. (2010) 37:463–70. doi: 10.1111/j.1346-8138.2010.00804.x 20536652

[37] Gaillat J Gajdos V Launay O Malvy D Demoures B Lewden L . Does monastic life predispose to the risk of Saint Anthony's fire (herpes zoster)? Clin Infect Dis. (2011) 53:405–10. doi: 10.1093/cid/cir436 21844022

[38] Johnson BH Palmer L Gatwood J Lenhart G Kawai K Acosta CJ. Annual incidence rates of herpes zoster among an immunocompetent population in the United States. BMC Infect Dis. (2015) 15:502. doi: 10.1186/s12879-015-1262-8 26546419 PMC4636742

[39] Ragozzino MW Melton LJ Kurland LT Chu CP Perry HO. Population-based study of herpes zoster and its sequelae. Medicine. (1982) 61:310–6. doi: 10.1097/00005792-198209000-00003 6981045

[40] Levin MJ Smith JG Kaufhold RM Barber D Hayward AR Chan CY . Decline in varicella-zoster virus (VZV)-specific cell-mediated immunity with increasing age and boosting with a high-dose VZV vaccine. J Infect. (2003) 188:336–44. doi: 10.1086/379048 14593591

[41] Tang H Moriishi E Okamoto S Okuno Y Iso H Asada H . A community-based survey of varicella-zoster virus-specific immune responses in the elderly. J Clin Virol. (2012) 55:46–50. doi: 10.1016/j.jcv.2012.06.008 22771002

[42] Choo PW Galil K Donahue JG Walker AM Spiegelman D Platt R. Risk factors for postherpetic neuralgia. Arch Intern Med. (1997) 157:1217–24. doi: 10.1001/archinte.1997.00440320117011 9183233

[43] Dworkin RH Portenoy RK. Pain and its persistence in herpes zoster. Pain. (1996) 67:241–51. doi: 10.1016/0304-3959(96)03122-3 8951917

[44] Meister W Neiss A Gross G Doerr H Höbel W Malin J . Demography, symptomatology, and course of disease in ambulatory zoster patients. A physician-based survey in Germany. Intervirology. (1998) 41:272–7. doi: 10.1159/000024949 10325537

[45] Forbes HJ Thomas SL Smeeth L Clayton T Farmer R Bhaskaran K . A systematic review and meta-analysis of risk factors for postherpetic neuralgia. Pain. (2016) 157:30–54. doi: 10.1097/j.pain.0000000000000307 26218719 PMC4685754

[46] Thomas SL Hall AJ. What does epidemiology tell us about risk factors for herpes zoster? Lancet Infect Dis. (2004) 4:26–33. doi: 10.1016/S1473-3099(03)00857-0 14720565

[47] Drolet M Brisson M Levin MJ Schmader KE Oxman MN Johnson RW . A prospective study of the herpes zoster severity of illness. Clin J Pain. (2010) 26:656–66. doi: 10.1097/AJP.0b013e3181eef686 20842005

[48] Schmader K. Herpes zoster in older adults. Clin Infect Dis. (2001) 32:1481–6. doi: 10.1086/320169 11317250

[49] Johnson R. Herpes zoster-predicting and minimizing the impact of post-herpetic neuralgia. J Antimicrob Chemother. (2001) 47:1–8. doi: 10.1093/jac/47.suppl_1.1 11160029

[50] Johnson RW. Zoster-associated pain: what is known, who is at risk and how can it be managed? Herpes. (2007) 14 (Suppl2):30–4. 17939893

[51] Dworkin RH Gnann JW Oaklander AL Raja SN Schmader KE Whitley RJ. Diagnosis and assessment of pain associated with herpes zoster and postherpetic neuralgia. J Pain. (2008) 9:S37–44. doi: 10.1016/j.jpain.2007.10.008 18166464

[52] Farahat F AlZunitan M Alsaedi A Al Nassir W Elgammal A Nazeer S . Epidemiology of herpes zoster in national guard hospitals in Saudi Arabia: a 6-year retrospective chart review study. Front Public Health. (2025) 12:1479640. doi: 10.3389/fpubh.2024.1479640 40051675 PMC11882511

[53] Yawn BP Wollan P St Sauver J. Comparing shingles incidence and complication rates form medical record review and administrative database estimates: how close are they? Am J Epidemiol. (2011) 174:1054–61. doi: 10.1093/aje/kwr206 21920944 PMC3243933

[54] Klompas M Kulldorff M Vilk Y Bialek SR Harpaz R. Herpes zoster and postherpetic neuralgia surveillance using structured electronic data. Mayo Clin Proc. (2011) 86:1146–53. doi: 10.4065/mcp.2011.0305 21997577 PMC3228613

[55] Zhou H Wang Z Jin H Chen X Lei L. A systematic review and meta-analysis of independent risk factors for postherpetic neuralgia. Ann Palliat Med. (2021) 10:12181–9. doi: 10.21037/apm-21-3028 35016460

[56] Stefanizzi P Moscara L Palmieri C Martinelli A Di Lorenzo A Scaltrito C . Safety profile and medium- to long-term protection of the recombinant zoster vaccine (RZV) in a cohort of high-risk patients: real-world data from a general hospital in Southern Italy, 2021-2025. Expert Rev Vaccines. (2025) 24:1059–68. doi: 10.1080/14760584.2025.2589216 41220243

[57] Zeevaert R Thiry N Maertens de Noordhout C Roberfroid D. Efficacy and safety of the recombinant zoster vaccine: a systematic review and meta-analysis. Vaccine X. (2023) 15:100397. doi: 10.1016/j.jvacx.2023.100397 37867572 PMC10589374

[58] Marra F Yip M Cragg JJ Vadlamudi NK. Systematic review and meta-analysis of recombinant herpes zoster vaccine in immunocompromised populations. PLoS ONE. (2024) 19:e0313889. doi: 10.1371/journal.pone.0313889 39585863 PMC11588208

[59] Bakacs T Sandig V Kovesdi I. Combination therapy for the treatment of shingles with an immunostimulatory vaccine virus and acyclovir. Pharmaceuticals. (2023) 16:226. doi: 10.3390/ph16020226 37259374 PMC9958712

[60] Forbes HJ Bhaskaran K Thomas SL Smeeth L Clayton T Mansfield K . Quantification of risk factors for postherpetic neuralgia in herpes zoster patients: a cohort study. Neurology. (2016) 87:94–102. doi: 10.1212/WNL.0000000000002808 27287218 PMC4932239

